# Pilonidal sinus (*Nadi vrana*): A case study

**DOI:** 10.4103/0974-7788.72492

**Published:** 2010

**Authors:** Pradeep Shinde, Hemant Toshikhane

**Affiliations:** *Department of Shalayatantra, KLEU Shri B.M.K Ayurveda Mahavidyalaya, Shahapur, Belgaum, Karnataka, India*

**Keywords:** *Kshara*, *Ksharasutra*, *ksharavarti*, *nadi vrana chikitsa*, pilonidal sinus

## Abstract

Pilonidal sinus (PNS) occurs in the cleavage between the buttocks (natal cleft) and can cause discomfort, embarrassment and absence from work for thousands of young people (mostly men) annually. The incidence of the disease is calculated to be 26 per 100,000 people. It occurs 2.2 times more often in men than in women. Age at presentation is 21 years for men and 19 years for women this case report describes a 22-year-old man with pilonidal sinus who was treated with *ksharasutra*.

## INTRODUCTION

Pilonidal sinus (Pilonidal disease was first described by Hodges in 1880) occurs in the cleavage between the buttocks (natal cleft) and can cause discomfort, embarrassment and absence from work for thousands of young people (mostly men) annually. The incidence of the disease is calculated to be 26 per 100,000 people. It occurs 2.2 times more often in men than in women. Age at presentation is 21 years for men and 19 years for women.[1] It is a common problem in primary care due to recurrence following surgery and the need for frequent and time-consuming wound care. This disorder is characterized by a characteristic epithelial track (the sinus) situated in the skin of the natal cleft, a short distance behind the anus and generally containing hair. Hence, the name ‘pilonidal’ is given, which is derived from Latin literally meaning ‘nest of hairs’. During the Second World War, the condition was common in jeep drivers, which led to it being known as ‘jeep disease’. A similar condition arises in the clefts between the fingers of barbers or hairdressers caused by customers’ hair entering moist, damaged skin.

In Ayurveda texts, no direct reference to pilonidal sinus (PNS) as a disease entity is found. However, Sushruta has described that hair can be a root cause.[2] for the formation of a sinus, and also mentioned various methods of management including *agnikarma* and *ksharasutra*^3^

## CASE REPORT

A 22-year-old boy approached us with complaints of pain and pus discharge from the anal verge, with intermittent fever since 2 years. On local examination, a scar measuring 4 cm at 5 O’clock position was found with two external openings on either end of the scar, at about 9 cm away from anal verge.

Routine investigations were within normal limits. Intraoperatively, a dye was injected through the external opening to see if there was any connection with the anus. It was seen that the dye was coming from the natal cleft which confirmed a diagnosis of PNS and not *fistula in ano*. An elliptical incision was taken over the natal cleft till the pre-sacral fascia to excise the sinus, and a bunch of hair was removed. The wound was allowed to heal by secondary intention. The remaining tract measuring about 9 cm, extending from base of natal cleft to the external opening over the scar at 5-o’clock position, was threaded with *Apamarg Ksharasutra*. The ramifications and communicants with this tract were treated with *ksharavartiy* (alkali suppositories).

The postoperative course was uneventful. The *Apamarg Ksharasutra* was changed every fourth day, on day care basis, and this process was continued till the complete cutting of the tract. Complete healing occurred after four weeks.

## DISCUSSION

The commonly adopted surgical techniques in contemporary science for management of PNS include incision and drainage, excision and healing by secondary intention, excision and primary closure, and excision with reconstructive flap techniques. In the present case, as the external openings were far away from the natal cleft and many ramifications were present, excision and healing by secondary intention was adopted. *Kshara* (alkali) and its different modalities like *ksharavarti, Ksharasutra*, etc. are unique contributions of ancient science. As *Ksharasutra* exerts both cutting and healing actions, it was used for threading between the main sinus and the external opening which was approximately 9 cm.

It is an accepted fact that average unit cutting time with *Ksharasutra* is 1 cm/week in *fistula-in-ano* and the *ksharasutra* is therefore changed once a week. In our case we have changed the *ksharasutra* every fourth day. The unit cutting time depends on various factors like the concentration of the drug, tissue of the tract and pressure applied by the *ksharasutra* on the tract. In this case, since we changed the *ksharasutra* every fourth day, good pressure was exerted on the sinus. It was considered appropriate to reduce the time to 4 days as it was not an anal fistula, and there was less chance of contamination of the tract since it was not connected to the anal canal.

Pilonidal disease is a complex condition that causes both discomfort and embarrassment to sufferers, and imposes direct costs to the healthcare system and indirect costs to society through absence from work. Regardless of the surgical technique concerned, standard principles of wound care are essential with repeated depilation of the natal cleft, removal of hair and any debris from the wound bed and keeping the wound edges separated using an appropriate dressing.
